# Indicators of early transfusion in paediatric trauma: a retrospective analysis of 11,849 cases from the TraumaRegister DGU^®^

**DOI:** 10.1186/s13049-025-01516-x

**Published:** 2025-11-26

**Authors:** Niko R. E. Schneider, Ralf Kraus, Rolf Lefering, Fabian Hemm, Davut Deniz Uzun, Christan Heiss, Andreas Hecker, Michael Sander, TraumaRegister DGU®, Emmanuel Schneck

**Affiliations:** 1 Department of Anesthesiology, Operative Intensive Care Medicine and Pain Therapy, University Hospital of Giessen, Justus-Liebig-University of Giessen, Giessen, Germany; 2 Department of Trauma-, Hand- and Reconstructive Surgery, University Hospital of Giessen, Justus-Liebig-University of Giessen, Giessen, Germany; 3 Institute for Research in Operative Medicine (IFOM), University Witten/Herdecke, Cologne, Germany; 4 Medical Faculty Heidelberg, Department of Anesthesiology, Heidelberg University, Heidelberg, Germany; 5 Department of General, Thoracic and Transplant Surgery, University Hospital of Giessen, Justus-Liebig-University of Giessen, Giessen, Germany

**Keywords:** Children, Red blood cell concentrate, Haemorrhage, Paediatric trauma, Emergency medicine

## Abstract

**Background:**

The transfusion of red blood cell (RBC) concentrates represents an emerging approach in paediatric trauma management, even in the prehospital period. Nevertheless, distinctive parameters for predicting the need for transfusion in children are still lacking. This study aimed to identify indicators for early in-hospital RBC transfusions that are primarily accessible either in the prehospital or in the early in-hospital setting in a large paediatric trauma cohort.

**Methods:**

This study comprised a retrospective analysis of the German TraumaRegister DGU^®^. It included children and adolescents aged 1 to 16 years from Germany, Austria, and Switzerland over a 15-year period. Contingency tables were used to identify risk factors, which were then assessed through multivariate regression analysis. The model’s predictive capacity was evaluated using the receiver operating characteristic (ROC) curve.

**Results:**

A total of 11,849 patients were included, with RBC transfusion performed in 5.9% of cases. Polytraumatised patients (adjusted odds ratio (adj. OR) 4.18 [95% confidence interval 3.26–5.34]) and those with penetrating injuries (adj. OR 4.32 [2.96–6.30]) and abdominal injuries (adj. OR 4.18 [3.34–5.24]) exhibited the highest risk of requiring an RBC transfusion. The need for cardiopulmonary resuscitation (adj. OR 2.46 [1.84–3.28]), endotracheal intubation (adj. OR 2.51 [1.93–3.28]), and Glasgow Coma Scale (GCS) ≤ 8 (adj. OR 2.49 [1.85–3.36]) were also significant, but weaker, predictors. A model based on the mentioned parameters achieved an area under the ROC curve of 0.87 [0.85–0.88], whereas predictive performance was lower but still acceptable when only parameters available in the prehospital setting were included (AUC 0.80 [0.78–0.82]).

**Conclusion:**

The likelihood of requiring an RBC transfusion is increased in cases of polytrauma, abdominal and penetrating trauma, patients with a GCS ≤ 8, and those requiring tracheal intubation or cardiopulmonary resuscitation. Therefore, the proposed risk factors can help identify patients at risk of severe haemorrhage and subsequent transfusion requirement.

**Clinical trial number:**

Not applicable.

**Graphical Abstract:**

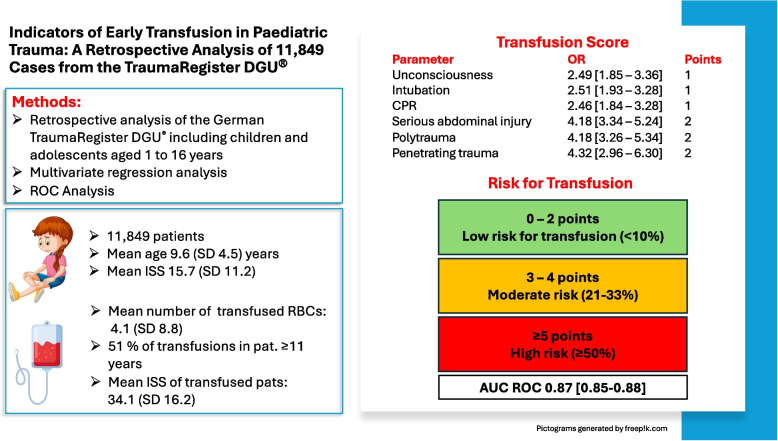

**Supplementary Information:**

The online version contains supplementary material available at 10.1186/s13049-025-01516-x.

## Background

Trauma remains the leading cause of death in children in Europe and the United States (US) [1, 2]. The main factors contributing to this high mortality rate are traumatic brain injuries, hypoxia, and haemorrhage, which rapidly worsen the patient’s condition and often result in death at the scene [1, 2]. An analysis of the National Trauma Data Bank of the US on the temporal distribution of death found that 51% of paediatric patients were already deceased upon arrival, highlighting the need for urgent treatment at the scene [3].

Considering causes of death, as delineated by the cABCDE algorithm, emergency medical services (EMS) should address the most urgent issues in paediatric trauma, that is, immediate critical bleeding control, securing the airway and providing adequate ventilation, volume resuscitation, and minimising on-scene time, to enable timely surgical treatment and transfusion [4]. The transfusion of red blood cell (RBC) concentrates, combined with the replacement of coagulation factors and platelets, remains an essential component of trauma resuscitation in the emergency room [5].

Early transfusion plays a pivotal role in paediatric trauma resuscitation, possibly even before a massive transfusion protocol needs to be applied. Derived from military experience, the prehospital transfusion (PT) of RBCs represents the maximal variant of early transfusion and has been introduced into civilian EMS [6]. Increasing evidence supports PT in adult populations based on reduced trauma-associated morbidity and mortality; thus, it has been implemented in European EMS [7]. A survey by the European Society of Anaesthesiology revealed that 48% of respondents, primarily from helicopter EMS, had the logistical capacity to perform PT [8]. PT’s technical feasibility has also been proven previously [9]. Recently, the combined use of RBC and plasma was shown to improve survival in adult trauma patients [10, 11]. Only limited data on PT in children are available. However, in 2023, a landmark study by Morgan et al. investigated 559 children from the Pennsylvania Trauma Systems Foundation database, 13% of whom received PT. The study showed that early transfusion at the scene resulted in reduced 24-h and in-hospital mortality compared to early in-hospital transfusion [12]. Therefore, also the PT of RBCs should be considered in critically injured children.

In addition to establishing the logistics for PT, determining the correct indication remains a major challenge for emergency response teams, regardless of whether transfusion occurs in the prehospital or early in-hospital phase. On one hand, unnecessary transfusions must be avoided; on the other hand, restrictive approaches might lead to further deterioration of the circulation. Trauma teams face a time-critical situation and only have information on the patient’s current status to decide if a transfusion should be performed. Data derived from adult populations suggest indicators for coagulopathy, such as base excess and lactate levels, as predictors for massive transfusion. Often, these are not available in the early phase of trauma management [13, 14]. In general, data on predictors for trauma-associated transfusions in children are limited [15]. Therefore, the present analysis aimed to identify predictors for early in-hospital transfusion that are primarily accessible (or can be reasonably approximated) in the prehospital or in the early in-hospital setting in a large paediatric trauma cohort.

## Methods

### Study design

A retrospective analysis of the German TraumaRegister DGU^®^ of the German Trauma Society (Deutsche Gesellschaft für Unfallchirurgie (DGU)) was performed. As it involved analyses of routine, anonymous data, no ethical approval was necessary (Justus Liebig University Giessen, Giessen, Germany; correspondence from February 25, 2025). The study followed the current publication guidelines of the TraumaRegister DGU^®^ and was registered under the TR-DGU Project ID 2024–040. The study was conducted according to the principles of the Declaration of Helsinki [16]. The methods and results are presented according to the Strengthening the Reporting of Observational Studies in Epidemiology (STROBE) guidelines [17].

### TraumaRegister DGU^®^

The TraumaRegister DGU^®^ of the German Trauma Society was founded in 1993. This multicentre database aims for the pseudonymised and standardised documentation of severely injured patients. Data are collected prospectively in four consecutive time phases from the site of the accident until discharge from the hospital: A) prehospital phase, B) emergency room and initial surgery, C) intensive care unit (ICU), and D) discharge. The documentation includes detailed information on demographics, injury pattern, comorbidities, pre- and in-hospital management, course in the intensive care unit, relevant laboratory findings (including data on transfusion), and the outcome of each individual. The inclusion criterion is hospital admission via the emergency room with subsequent ICU care or arrival at the hospital with vital signs and death before ICU admission.

The infrastructure for documentation, data management, and data analysis is provided by the Academy for Trauma Surgery (Akademie der Unfallchirurgie GmbH), a company affiliated with the German Trauma Society. The scientific leadership is provided by the Committee on Emergency Medicine, Intensive Care and Trauma Management (Sektion NIS) of the German Trauma Society. The participating hospitals submit their pseudonymised data to a central database via a web-based application. Scientific data analysis is approved according to a peer review procedure in the publication guidelines of TraumaRegister DGU^®^. The participating hospitals are primarily located in Germany (90%), but a rising number of hospitals from other countries also contribute data (currently, Austria, Belgium, China, Finland, Luxembourg, Slovenia, Switzerland, the Netherlands, and the United Arab Emirates). Currently, about 38,000 cases from almost 700 hospitals are entered into the database per year. Participation in TraumaRegister DGU^®^ is voluntary. For hospitals associated with TraumaNetzwerk DGU^®^, however, the entry of at least a basic dataset is obligatory for quality assurance purposes.

### Inclusion criteria

The study included patients from Germany, Austria, and Switzerland over a 15-year period (January 1, 2008, to December 31, 2023). Only children and adolescents aged 1 to 16 years were included. Transfers between hospitals to higher-level care centres were excluded. Only data from the core collective were analysed. To exclude patients with minor injuries, only those with a Maximal Abbreviated Injury Scale (MAIS) score ≥ 3 or those with a MAIS score ≥ 2 who required ICU treatment or died were included.

### Definition of massive transfusion

The definition of massive transfusion in paediatric patients is not uniform. In line with commonly applied criteria, massive transfusion was defined as the transfusion of blood products estimated at least one blood volume within 24 h or half a blood volume within 12 h [18, 19]. As individual body weights were not available in the TraumaRegister DGU^®^ and RBC unit volumes vary, we derived pragmatic, age-specific thresholds for the number of RBC units corresponding to the following definitions: 2 RBC units for the age of 1–2 years; 3 RBC units for the age of 3–4 years; 4 RBC units for the age of 5–6 years; then increase by 1 RBC unit per year until reaching 10 units at age 12 (the commonly used adult threshold for massive transfusion).

### Statistical analysis

Descriptive analysis of the study cohort was performed, with data presented as absolute numbers and percentages. Metric data are presented as mean with standard deviation (SD). Parameters included baseline characteristics, as well as details regarding the severity and location of injuries. An Abbreviated Injury Scale (AIS) score ≥ 3 was classified as a serious injury, while an Injury Severity Score (ISS) ≥ 16 was considered indicative of severe multiple trauma. Polytrauma was defined according to the Berlin definition as injuries affecting at least two body regions with an AIS score ≥ 3, accompanied by at least one physiological deterioration parameter (systolic blood pressure ≤ 90 mmHg, Glasgow Coma Scale ≤ 8, base excess ≤ − 6.0 mmol/L, coagulopathy (INR ≥ 1.4 or PTT ≥ 40 s), or body temperature ≤ 34 °C) [20]. High-energy trauma was defined as injuries resulting from motor vehicle collisions, motor bike accidents, or falls from a height of ≥ 3 m.

The chi-square test was used for categorical data, comparing patients with and without transfusions; metric data were compared with the Mann–Whitney U-test. A *p*-value < 0.05 was considered statistically significant.

Finally, a multivariate logistic regression analysis with transfusion as the dependent variable was performed, including potential predictors from the univariate analysis. Since several parameters differed significantly between patients with and without transfusion, trauma experts assessed if they were clinically present for EMS at the emergency scene or only in the early in-hospital phase before validation in the multivariate regression analysis. This resulted in two prediction models: one for the prehospital phase and one for the early in-hospital phase (trauma room), with the latter including AIS and ISS. The results are presented as adjusted odds ratios (ORs) with 95% confidence intervals. Variables with an OR > 2.0 were used to build a simple point score: 1 point if the OR was 2.0–3.0 and 2 points if the OR was > 3.0. Receiver operating characteristic (ROC) curve analysis was then performed to assess the predictive accuracy of the score. The area under the ROC curve was presented with 95% confidence intervals.

Statistical analyses were conducted using SPSS (version 29, IBM Inc., Armonk, NY, US).

## Results

### Study cohort

**Fig. 1:**
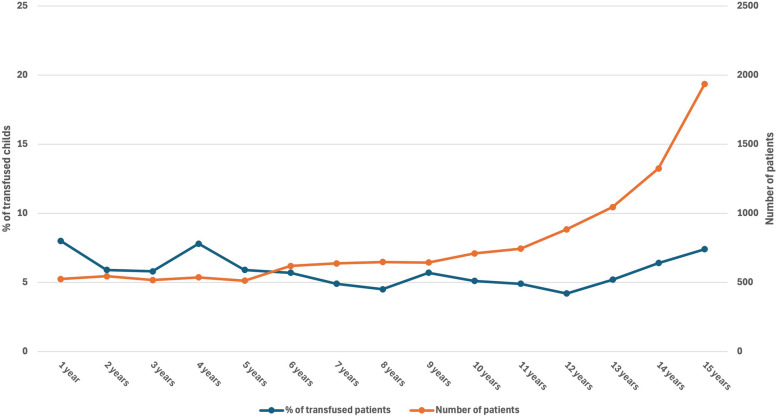
Graph showing the number of patients and the transfusion rate for each age

Overall, 11,849 patients of 569 hospitals were included in the study, with blood transfusions performed in 5.9% of cases. If patients were transfused, they received 4.1 (SD 8.8) RBC units on average. The transfusion rate remained relatively stable across different age categories, ranging from 4.2% in 12-year-olds to 8.0% in 1-year-olds (Fig. 1). The mean age of the included patients was 9.6 years (SD 4.5). However, the absolute number of transfusions increased significantly in older children and adolescents, who accounted for 51% of all transfusions (age ≥ 11–16 years, *n* = 358, *p* < 0.001).

According to the above-mentioned definitions, a massive transfusion was administered in 117 cases (0.99%) which assembles 16.7% of all transfused patients. The age distribution of cases with massive transfusion is shown in supplemental Table 1. In children, massive transfusion was associated with a significantly higher mean ISS (41.4 vs. 34.1 with transfusion and 14.5 without) and a higher mortality rate (64.1% vs. 36.8% and 2.8%, respectively).

**Table 1:** Basic characteristics and overview of underlying trauma mechanisms

Parameters	All patients (*n* = 11,849)	No transfusion (*n* = 11,147)	Transfusion (*n* = 702)	*p*-value
*Age (years)*				<.001
* Toddler [1–5 years], n (%)*	2,634 (22.2)	2,458 (22.1)	176 (25.1)	
* School child [6–10 years], n (%)*	3,256 (27.5)	3,088 (27.7)	168 (23.9)	
* Adolescents [11–15 years], n (%)*	5,959 (50.3)	5,601 (50.2)	358 (51.0)	
*Sex (n* = *11,838)*				.026
* Male, n (%)*	7,444 (62.9)	7,031 (63.1)	413 (58.9)	
*Died in hospital*	571 (4.8)	313 (2.8)	258 (36.8)	<.001
*Trauma mechanism*				<.001
* Motor vehicle accident, n (%)*	1,320 (11.3)	1,188 (10.8)	132 (19.1)	
* Motorcycle accident, n (%)*	736 (6.3)	685 (6.3)	51 (7.4)	
* Bicycle accident, n (%)*	1,833 (15.7)	1,750 (16.0)	83 (12.0)	
* Pedestrian accident, n (%)*	2,331 (20.0)	2,198 (20.1)	133 (19.2)	
* Fall from a height of* ≥ *3 m, n (%)*	2,043 (17.5)	1,899 (17.3)	144 (20.8)	
* Fall from a height of* < *3 m, n (%)*	1,632 (14.0)	1,602 (14.6)	30 (4.3)	
* Others, n (%)*	1,748 (15.0)	1,630 (14.9)	118 (17.1)	
*Severe trauma types*				
* High energy trauma*	4,099 (34.6)	3,772 (32.7)	327 (40.4)	<.001
* Traffic-associated accidents*	6,576 (55.5)	6,143 (55.1)	433 (61.7)	<.001
* Penetrating trauma*	334 (2.8)	276 (2.5)	58 (8.3)	<.001
* Violence-associated trauma*	156 (1.3)	129 (1.2)	27 (3.8)	<.001
*Level of hospital care*				
* Basic hospital care (Level 3)*	784 (6.6)	765 (6.9)	19 (2.7)	<.001
* Specialized hospital care (Level 2)*	3,008 (25.4)	2,903 (26.0)	105 (15.0)	<.001
* Comprehensive hospital care (Level 1)*	8,057 (68.0)	7.479 (67.1)	578 (82.3)	<.001
* Helicopter emergency medical service transportation*	3,335 (29.2)	3,040 (28.3)	295 (43.6)	<.001
*Trauma severity*				
* ISS* ≥ *16, n (%)*	5,048 (42.6)	4,405 (39.5)	643 (91.6)	<.001
* Polytrauma, n (%)*	995 (8.4)	630 (5.7)	365 (52.0)	<.001
* Single trauma, n (%)*	6,591 (55.6)	6,333 (58.8)	258 (36.8)	<.001
*Abbreviated injury scale*				
* AIS head* ≥ *3, n (%)*	4,321 (35.5)	3,901 (35)	420 (59.8)	<.001
* AIS thorax* ≥ *3, n (%)*	2,366 (20.0)	2,006 (18)	360 (51.3)	<.001
* AIS abdomen* ≥ *3, n (%)*	1,330 (11.2)	1,075 (9.6)	255 (36.3)	<.001
* AIS extremities* ≥ *3, n (%)*	2,678 (22.6)	2,390 (21.4)	288 (41.0)	<.001

Data are presented as absolute numbers and percentages. Data points were available in 11,643 cases for trauma mechanisms and in 11,423 for helicopter emergency medical service transportation, respectively. All other parameters were available in all included patients. Statistical significance refers to the difference between transfused and non-transfused patients per each parameter. *P*-values were calculated using the chi-square test

*AIS* abbreviated injury scale, *ISS* injury severity scale

The majority of patients were treated in level 1 or 2 hospitals, while only a small minority (< 1%) received transfusions in level 3 hospitals. The mean transportation time from scene to hospital was 59.9 min (SD 26.2, n = 9,185). Baseline characteristics and the underlying trauma mechanisms are shown in Table 1.

### Injury severity

In total, 42.6% of patients suffered from severe trauma, defined as an ISS ≥ 16 (mean ISS 15.7, SD 11.2; *n* = 11,849), while only 8.4% met the Berlin definition of polytrauma. The risk of death based on the Revised Injury Severity Scale II (RISC II) was 34.7% (*n* = 702) and 3.4% (*n* = 11,147) in transfused and non-transfused patients, respectively [21]. The mean ISS of non-transfused patients was 14.5 (SD 9.7), whereas transfused patients had a significantly higher mean ISS of 34.1 (SD 16.2; *p* < 0.01). While children with polytrauma (Berlin definition) reached the highest specificity for transfusion (94.3%), the sensitivity remained low (52%). The ISS offered a low specificity (60.5%) but a high sensitivity (91.6%). Overall, with the exception of an ISS ≥ 16 (positive predictive value (PPV) 63.2%), the PPV of single parameters remained low (Table 2). The highest transfusion rates were observed in children with head and thoracic injuries, followed by extremity and abdominal injuries (Table 1).

**Table 2:** Prevalence, specificity, sensitivity, and positive predictive value (PPV) for various clinical predictors of transfusion

Parameters	Prevalence (n, %)	Specificity (%)	Sensitivity (%)	PPV (%)
***Trauma severity***				
* ISS* ≥ *16*	5,048 (42.6)	60.5	91.6	63.2
* Polytrauma*	995 (8.4)	94.3	52.0	45.6
* Isolated injury*	6,591 (55.6)	43.2	36.8	44.8
* Penetrating trauma*	334 (2.8)	97.5	8.3	17.4
* Violence-associated trauma*	156 (1.3)	98.8	3.8	17.3
* High energy trauma*	4099 (34.6)	66.2	46.6	8.0
* Traffic-associated accidents*	6576 (55.5)	44.9	61.7	6.6
***Injury pattern (AIS* ≥ *3)***				
* Serious head injury*	4321 (36.5)	65.0	59.8	9.7
* Serious thorax trauma*	2366 (20.0)	82.0	51.3	15.2
* Serious abdominal trauma*	1330 (11.2)	90.4	36.3	19.2
* Serious extremity injury*	2678 (22.6)	78.6	41.0	10.8
***Pre-clinical assessment and interventions***				
* Systolic blood pressure* ≤ *90 mmHg*	1266 (13.6)	88.1	42.7	17.6
* Glasgow Coma Scale 3–8*	1942 (18.0)	84.6	59.4	19.8
* Volume therapy*	8439 (74.8)	25.6	81.1	6.6
* Catecholamine therapy*^a^	434 (6.4)	95.9	33.5	40.3
* Cardiopulmonary resuscitation*	433 (3.8)	97.5	23.7	37.6
* Tracheal Intubation*	2668 (23.6)	79.2	67.8	17.5
* Emergency thoracocentesis*^a^	108 (1.6)	99.1	9.4	45.4

*AIS* abbreviated injury scale, *ISS* injury severity scale

^a^Not available in the reduced basic dataset

An AIS ≥ 3 did sufficiently discriminate between transfused and non-transfused patients, which was particularly evident for abdominal injuries. Most patients (63.7%) with abdominal lesions were categorised as AIS < 3. Nevertheless, robust statistical differences were observed, with significantly more transfused than non-transfused children across all AIS categories, ISS ≥ 16, polytrauma, and single trauma.

### Clinical status of patients and emergency procedures

In total, 33.9% of patients were not alert at the scene, of whom 15.9% had a Glasgow Coma Scale (GCS) of 9–13, and 18% had a GCS ≤ 8. Overall, in 23.7% of cases, the airway was secured via intubation. The transfusion rate was significantly higher in unresponsive patients (GCS 3–8), those with pathological pupil reactions, and those requiring intubation (Table 3). Patients with a GCS of 9–13 did not have an increased need for transfusion.

**Table 3:** Contingency tables on indices of the clinical appearance and performed emergency procedures

Parameters	No transfusion (*n* = 11,147)	Transfusion (*n* = 702)	*p*-value
***Clinical appearance***			
* Systolic blood pressure* ≤ *90 mmHg, n (%)*	1,043 (11.9)	223 (42.7)	< 0.001
* Glasgow Coma Scale*			< 0.001
* 14–15, n (%)*	6,956 (68.6)	176 (27.2)	
* 9–13, n (%)*	1,624 (16.0)	86 (13.3)	
* 3–8, n (%)*	1,558 (15.4)	384 (59.4)	
*Pupillary light responsiveness*			< 0.001
* Normal, n (%)*	7,095 (88.3)	273 (49.1)	
* Delayed, n (%)*	605 (7.5)	95 (17.1)	
* None, n (%)*	333 (4.1)	188 (33.8)	
*Pupillary size*			< 0.001
* Regular size, n (%)*	10,052 (92.8)	434 (63.8)	
* Anisocoria, n (%)*	343 (3.2)	62 (9.1)	
* Bilaterally dilated, n (%)*	438 (4.0)	184 (27.1)	
***Emergency procedures***			
* Volume therapy, n (%)*	7,882 (74.4)	557 (81.1)	< 0.001
* Catecholamine therapy, n (%)*	259 (4.1)	175 (33.5)	< 0.001
* Cardiopulmonary resuscitation, n (%)*	270 (2.5)	163 (23.7)	< 0.001
* Tracheal Intubation, n (%)*	2,202 (20.8)	466 (67.8)	< 0.001
* Emergency thoracocentesis, n (%)*	59 (0.9)	49 (9.4)	< 0.001

Data are presented as absolute numbers and percentages. Volume therapy was defined as the administration of at least one unit of fluid (500 mL of crystalloids or colloids). Data was available for Glasgow coma scale in 10,784 cases, for pupillary light responsiveness in 8,589 cases, for pupillary size in 10,486 cases, volume therapy 6,775 cases, catecholamine therapy in 6,775 cases, cardiopulmonary resuscitation in 11,286 cases, tracheal intubation in 11,286 cases and thoracocentesis in 6,775 cases. All other parameters were available in all included patients. Statistical significance refers to the difference between transfused and non-transfused patients per each parameter. P-values were calculated using the chi-square test based on contingency tables

Regardless of age, a systolic blood pressure ≤ 90 mmHg was associated with a higher need for transfusion. At the scene, the mean systolic blood pressure was 115 mmHg (SD 26, *n* = 9,278), and the mean heart rate was 102 bpm (SD 27, *n* = 6,448). These vital signs did not differ at hospital admission (systolic blood pressure: 117 mmHg (SD 24); heart rate: 101 bpm (SD 25). No information on capillary refill time was available.

In total, patients received an average of 464 mL of fluids at the scene (SD 453, *n* = 11,286) and 768 mL in the resuscitation room (SD 1027, *n* = 5,772). Patients requiring transfusion received significantly more fluids (scene: 753 mL (SD 779) vs. 446 mL (SD 417); hospital: 2184 mL (SD 2160) vs. 640 mL (SD 731); *p* < 0.001).

### Risk prediction

The final multivariate logistic regression model included 10,594 patients of the 11,849 included patients because only 10,594 had complete data for all variables included in the regression model. The included parameters of the regression model are shown in Table 4.

**Table 4:** Results of the multivariate logistic regression analysis

Parameters	Adj. Odds ratio	95%-confidence interval	Weighting
*Injury severity*			
* Polytrauma*	4.18	3.26–5.34	2
* Thoracic AIS* ≥ *3*	1.19	0.95–1.49	
* Abdominal AIS* ≥ *3*	4.18	3.34–5.24	2
*Glasgow Coma Scale*			
* 9–13*	1.38	1.03–1.86	
* 3–8*	2.49	1.85–3.36	1
*Type of injury*			
* Penetrating*	4.32	2.96–6.30	2
* High energy trauma*	1.55	1.28–1.87	
*Emergency procedures*			
* Endotracheal Intubation*	2.51	1.93–3.28	1
* Cardiopulmonary resuscitation*	2.46	1.84–3.28	1

*AIS* Abbreviated injury scale

**Fig. 2:**
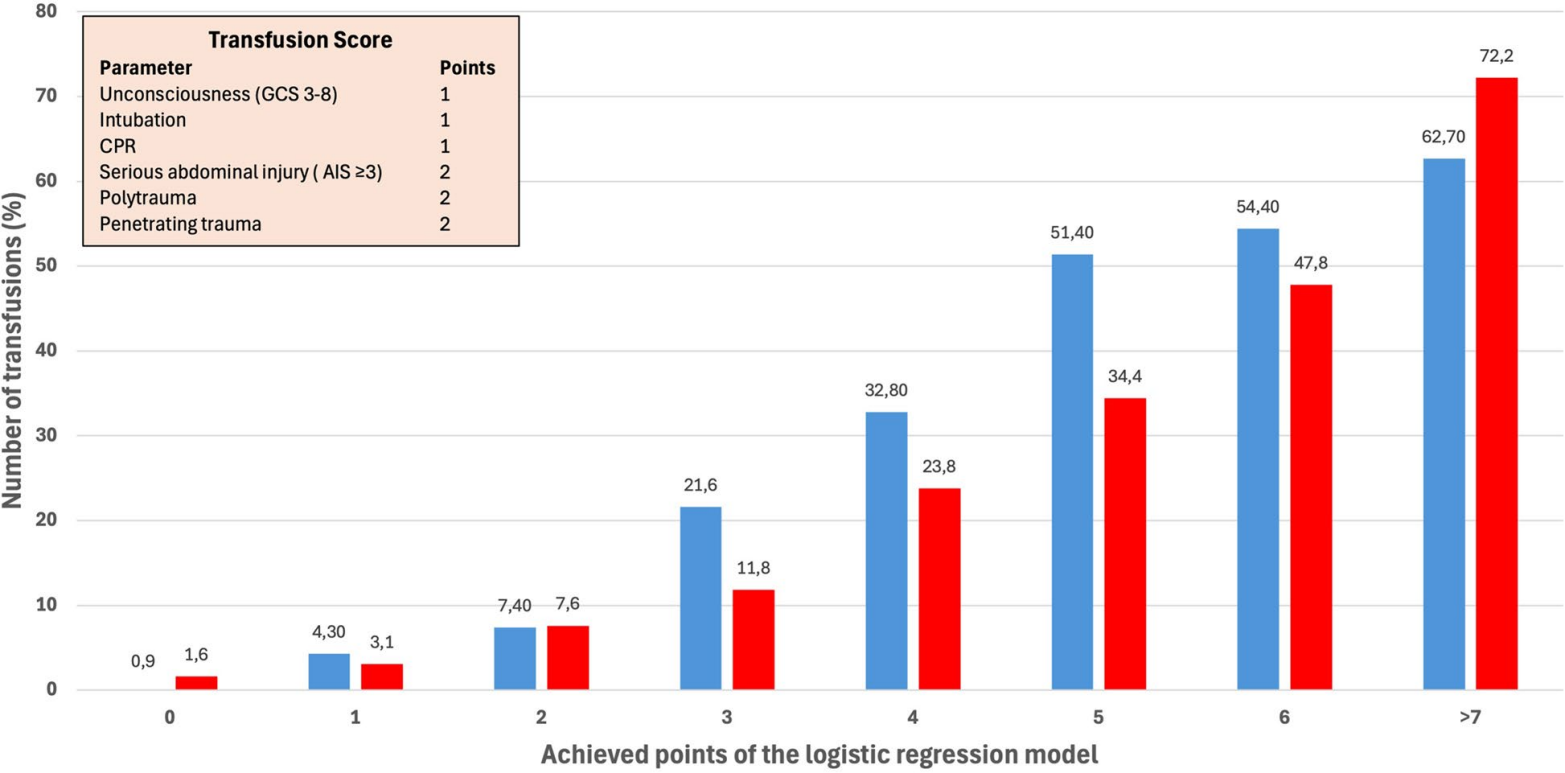
Cumulative transfusion rate based on the scoring point derived from the logistic regression analysis. The blue bars demonstrate the predictive power of all significant parameters while the red bars show only the results of the parameters available at the scene of emergency (penetrating injury, prehospital need for intubation, prehospital need for resuscitation, GCS 3–8). *Abbreviation: AIS* = *Abbreviated injury scale; CPR* = *Cardiopulmonary resuscitation*

The transfusion rate increased significantly from 7.4% (2 points) to 21.6% when 3 points were achieved (Fig. 2). If 5 or more points were reached, the transfusion rate exceeded 50%. In the model restricted to prehospital parameters, the transfusion probability was lower but increased also consistently with higher score points, from 1.6% at 0 points to 72.2% at 7 points (Fig. 2), indicating a stepwise relationship between the number of positive predictors and transfusion risk.

**Fig. 3:**
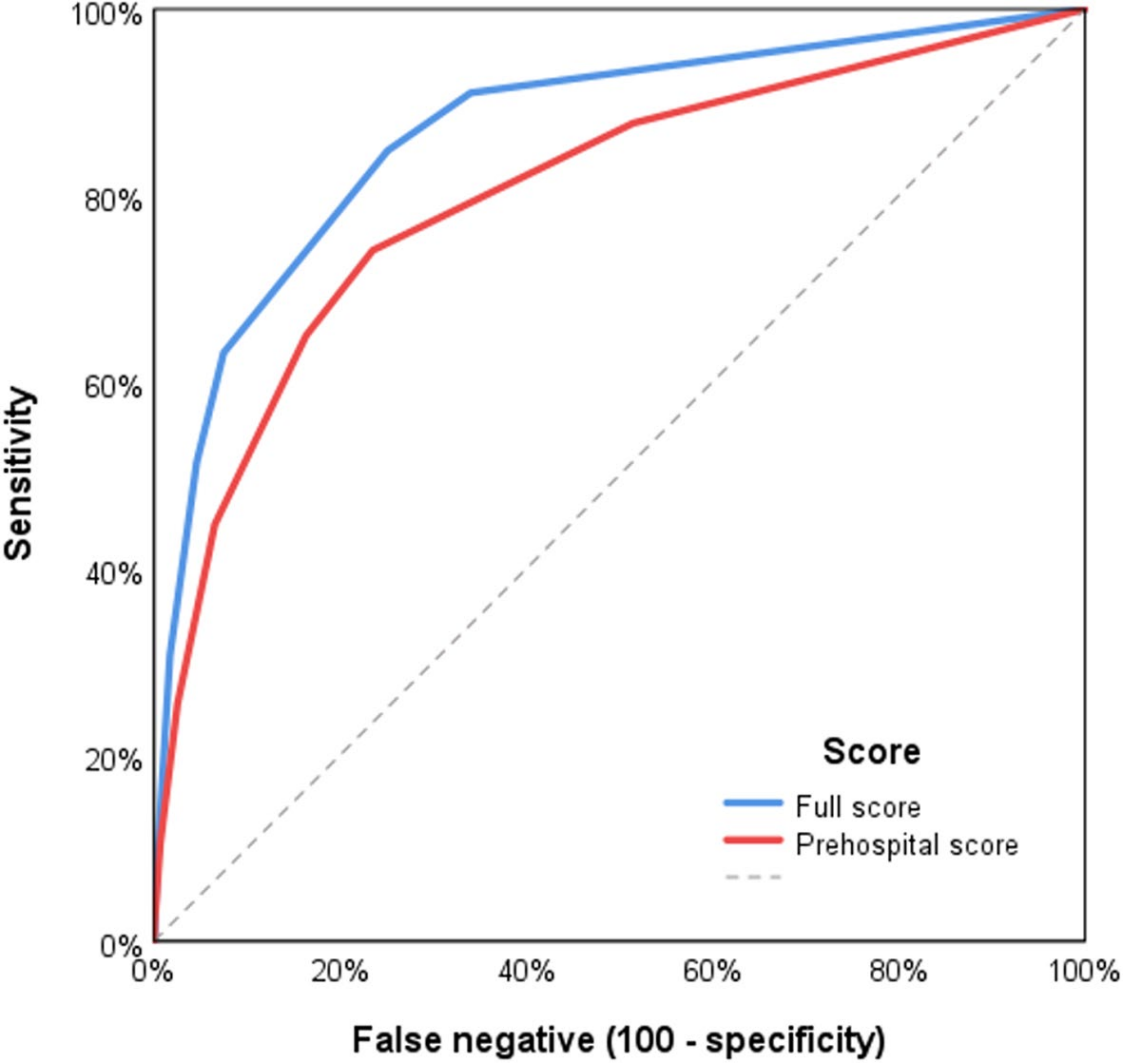
Results to the ROC analysis for the prediction of an intrahospital transfusion. The blue line demonstrates the predictive power of all significant parameters while the red line shows only the results of the parameters available at the scene of emergency (penetrating injury, prehospital need for intubation, prehospital need for resuscitation, GCS 3–8)

ROC analysis of all parameters with significant predictive power yielded an AUC of 0.87 [0.85–0.88] for transfusion prediction, whereas predictive performance was lower but still acceptable when only parameters available in the prehospital setting were included (AUC 0.80 [0.78–0.82], Fig. 3). In the latter model, all AIS-related parameters and the variable “polytrauma according to the Berlin definition” were excluded.

## Discussion

This study reviewed several predictors of early in-hospital transfusions in a large paediatric trauma cohort of 11,849 patients. It demonstrated that early assessment of clinically apparent parameters can sufficiently predict the need for transfusion in children. The highest risk for transfusion was observed in patients with penetrating trauma, abdominal injuries, and polytrauma, followed by those who were unconscious, required tracheal intubation, or underwent cardiopulmonary resuscitation.

A notable strength of this study lies in the high number of patients included, a crucial consideration given the limited number of paediatric trauma cases where transfusion was needed and the paucity of prospective studies in this domain. The observed transfusion rate is consistent with data from the US National Trauma Databank, which reported a transfusion rate of 4% within the first 24 h after hospital admission [22]. Like the data presented, a higher absolute number of transfusions was observed in older children and adolescents, severely injured patients (ISS ≥ 25), and unresponsive patients, making the results comparable to those in the US population.

While massive transfusion represents a clinically relevant endpoint in trauma resuscitation, its occurrence in paediatric trauma remains rare [18, 19]. In our cohort, only 117 (0.99%) patients met commonly used massive transfusion thresholds, corresponding to approximately eight cases per year. This low number precluded robust statistical modelling and underscores why focusing exclusively on massive transfusion would not adequately capture the clinical reality of paediatric trauma care. Moreover, the stepwise increase in mortality from non-transfused patients (2.8%) to transfused patients (36.8%) and those requiring massive transfusion (64.1%) highlights that transfusion itself, not only massive transfusion, is a strong marker of injury severity and outcome. This study therefore focused on identifying indicators of early transfusion rather than massive transfusion. Early recognition of transfusion needs may enable timely activation of haemostatic strategies, particularly in prehospital settings where full massive transfusion protocols are rarely feasible.

Several studies have investigated diagnostic algorithms for predicting transfusions in adults, resulting in the development of more than 20 scoring tools [23, 24]. In contrast, transfusion prediction in paediatric trauma care has been studied less. To date, only one other large registry study, an analysis of the Trauma Quality Improvement Project (TQIP), has aimed to identify intra- and prehospital predictors for transfusion after paediatric trauma [15]. This study used a Bayesian Belief Network to predict the probability of in-hospital transfusions in severely injured children. The transfusion rate in the TQIP study was lower than in the present analysis, with 2.8% of patients receiving a transfusion within the first 4 h after hospital admission. The model included 14 parameters and demonstrated a high predictive power for transfusion. The most influential predictors were GCS and impaired systolic blood pressure at admission and at the scene. This finding is particularly relevant because systolic blood pressure was not adjusted for age, indicating that a blood pressure ≤ 90 mmHg is predictive of transfusion regardless of age, aligning with our study. However, this finding should be interpreted with caution as 50% of the patients in our study were older than 10 years, and 27% were aged between 5 and 10 years. This suggests that while hypotension may be predictive of the need for transfusion in school-aged children and adolescents, it may not necessarily apply to younger children. This may also account for the significantly higher transfusion rate in this study, despite these factors not being independent predictors of transfusion, possibly since blood pressure and other vital signs were likely optimised by EMS personnel before hospital admission. Additionally, this might explain why heart rate failed to predict transfusion in our study and had only a minor impact in the TQIP study, especially when only prehospital vital signs were considered. Undisputably, age-adjusted vital parameters are ideal for the assessment of children’s hemodynamic status; however, these measurements are not practically achievable in the early phase of trauma resuscitation [25, 26]. Therefore, heart rate and blood pressure do not necessarily comprise important indicators of the need for transfusion, most likely because vital parameters were not analysed in an age-adjusted manner. Unfortunately, the capillary refill time, which could have been an easily assessable option, was not available in the TraumaRegister DGU^®^.

As early transfusion, including prehospital transfusion where feasible, may improve survival, we developed two predictive models reflecting parameters available at different stages of care: one focusing on predictors primarily accessible in the prehospital setting, and the second also incorporating parameters such as AIS and ISS that become available in the early in-hospital phase after radiographic diagnostics. Even though, severe injuries and multiple trauma accompanied by deterioration of vital signs can usually be recognised with sufficient accuracy by EMS, a calculation of the ISS (or AIS) is not possible in the pre-hospital setting. It should be noted that the prehospital teams involved in administering RBC transfusions commonly are not regular ground EMS units, but highly experienced advanced practitioners, such as helicopter EMS (HEMS) and specialised physician-staffed services. These teams are trained to recognise patterns of severe injury, and indicators such as low GCS or the need for intubation are strongly associated with both HEMS activation and a high likelihood of major head injury recognition. Nevertheless, studies have shown that only about half of abdominal injuries are correctly identified by HEMS, underlining the need for radiologic diagnostics [27, 28]. For this reason, abdominal AIS were only included in the in-hospital prediction model. Even though the model’s predictive power increased after adding abdominal AIS and an ISS ≥ 16, the prehospital parameters alone already provided acceptable predictive performance. In this context, it should be noted that the positive predictive value of individual parameters remains limited; however, their combination may allow for a clinically meaningful and reliable prediction of transfusion requirements.

Consistent with our findings, the TQIP registry showed that only the inclusion of impaired GCS improved the model’s predictive accuracy (AUC ROC 0.84 vs. 0.87 in our study), highlighting the importance of GCS assessment. On the other hand, abdominal injuries, the need for resuscitation, and the presence of multiple injuries did not improve the Bayesian model, whereas these factors were among the strongest predictors in our logistic model. Interestingly, the accident mechanism improved the predictive accuracy of the TQIP model, whereas the resulting injuries did not. This may be explained by the fact that the accident mechanism serves as an early proxy for injury severity, while the actual injuries often require time-consuming diagnostics. Since transfusion decisions must frequently be made before a complete assessment is available, the accident mechanism might have had a greater impact on the predictive model. Additionally, prehospital interventions could have mitigated the effects of some injuries, further reducing their predictive value. Finally, it is important to note that the TQIP analysis has not yet been externally validated.

Low GCS, tracheal intubation, and cardiopulmonary resuscitation showed a lower adjusted OR than the presence of polytrauma and abdominal and penetrating trauma. In our opinion, this reflects the underlying trauma pathophysiology. An impaired GCS is often caused by severe traumatic brain injury and the subsequent tracheal intubation and/or resuscitation [29]. On the other hand, multiple, penetrating, and abdominal traumas display common causes of severe bleeding [22, 24]. In summary, it is important to understand that with the exception of an ISS ≥ 16, the positive predictive value of individual parameters remains low, but their combination allows for a reliable prediction of transfusion needs. While some parameters were highly specific but had low sensitivity, the opposite was true for others. Moreover, the high prevalence of certain parameters (e.g., volume therapy) further reduced the positive predictive value. Therefore, each patient should be assessed based on multiple transfusion indicators rather than a single definitive criterion.

This study has several limitations. First and most importantly, it lacks a validation cohort. This is due to the low incidence of paediatric trauma requiring transfusion, which posed a challenge for the study design. A split-sample approach would have markedly reduced statistical power and rendered robust modelling impossible. This challenge is even more evident for massive transfusion, which is exceedingly rare in children. Additionally, data quality before 2008 was insufficient, and transfusion strategies may have changed over the past 25 years, making a historical validation cohort unfeasible. The use of data from other countries was also not a viable option, as only 5% of the TraumaRegister DGU^®^ data originates from outside Germany, Austria, and Switzerland. Therefore, the identified predictors must be regarded as hypothesis-generating and require external validation in independent cohorts before clinical application. To develop a reliable scoring tool, the identified risk factors must first be validated. Second, the point score cut-offs were derived post hoc from the observed ORs in our cohort. While this pragmatic approach allowed for the creation of a simple and intuitive tool, it may introduce a risk of overfitting. Therefore, the proposed score should be regarded as exploratory and requires external validation in independent datasets before clinical implementation. Third, children under 1 year of age were excluded due to limited data availability. However, most children were of school age or adolescents; therefore, the bias probably has little impact on the study results. Nevertheless, future studies should focus on neonates and infants, despite the rarity of these cases, to better understand their specific clinical outcomes. Fourth, only limited datasets were available for lactate and base excess, which have been identified as potential predictors of transfusion and could be assessed at the scene using point-of-care devices [13]. Fifth, this study did not report the amount of blood transfused relative to the patient’s body weight. Sixth, due to changes in documentation practices in 2020, with separate recording of transfusions in the trauma bay and operating room introduced only thereafter, a consistent phase-specific analysis across the full study period (2009–2023) was not methodologically feasible. Finally, due to the retrospective design, the investigators had to rely on the quality of the documentation within the database.

## Conclusion

In summary, based on a large cohort of 11,849 severely injured children and adolescents, this study demonstrated parameters which were predictive for an increased risk for intrahospital transfusion. When only parameters available in the prehospital phase were included, the predictive performance of the statistical model was acceptable, whereas the addition of more specific early in-hospital parameters such as abdominal AIS and polytrauma (Berlin definition) resulted in a highly significant model. This information aids in evaluating the early need for transfusion in these patients. An impaired GCS proved to be more predictive than other impaired vital parameters, such as hypotension or tachycardia, likely due to prior correction by EMS. Particularly in the case of penetrating trauma or trauma with need for resuscitation or endotracheal intubation, the probability of blood loss increases to such an extent that even the PT of RBC can be considered. Therefore, the proposed risk factors might help identify children at risk of an early transfusion requirement after trauma.

## Supplementary Information

Additional file 1. Supplemental Figure 1. Age distribution of cases with massive transfusion. Age is demonstrated as years. Abbreviations: pRBC: packed red blood cell concentrate

## Data Availability

The datasets used and/or analysed during the current study are available from the corresponding author on reasonable request.

## Abbreviations

Adj. OR: Adjusted odds ratio
AIS: Abbreviated Injury Scale
AUC: Area under the curve
DGU: Deutsche Gesellschaft für Unfallchirurgie
EMS: Emergency medical services
GCS: Glasgow Coma Scale
ICU: Intensive care unit
INR: International Normalised Ratio
ISS: Injury Severity Scale
MAIS: Maximal Abbreviated Injury Scale
PPV: Positive predictive value
PT: Prehospital transfusion
PTT: Partial Thromboplastin Time
RISC II: Revised Injury Severity Scale II
RBC: Red blood cell concentrate
ROC: Receiver operating characteristic
SD: Standard deviation
STROBE: Strengthening the Reporting of Observational Studies in Epidemiology
TQIP: Trauma Quality Improvement Project
US: United States

## References

[1] Drake SA, Holcomb JB, Yang Y, Thetford C, Myers L, Brock M, et al. Establishing a regional pediatric trauma preventable/potentially preventable death rate. Pediatr Surg Int. 2020. 10.1007/s00383-019-04597-9. 31701301 10.1007/s00383-019-04597-9

[2] Theodorou CM, Galganski LA, Jurkovich GJ, Farmer DL, Hirose S, Stephenson JT, et al. Causes of early mortality in pediatric trauma patients. J Trauma Acute Care Surg. 2021. 10.1097/TA.0000000000003045. 33492107 10.1097/TA.0000000000003045 PMC8008945

[3] McLaughlin C, Zagory JA, Fenlon M, Park C, Lane CJ, Meeker D, et al. Timing of mortality in pediatric trauma patients: a National Trauma Data Bank analysis. J Pediatr Surg. 2018. 10.1016/j.jpedsurg.2017.10.006. 29111081 10.1016/j.jpedsurg.2017.10.006 PMC5828917

[4] Freire GC, Beno S, Yanchar N, Weiss M, Stang A, Stelfox T, et al. Clinical practice guideline recommendations for pediatric multisystem trauma care: a systematic review. Ann Surg. 2023. 10.1097/SLA.0000000000005966. 37325908 10.1097/SLA.0000000000005966

[5] Rossaint R, Afshari A, Bouillon B, Cerny V, Cimpoesu D, Curry N, et al. The European guideline on management of major bleeding and coagulopathy following trauma: sixth edition. Crit Care. 2023;27:1–45. 10.1186/s13054-023-04327-7. 36859355 10.1186/s13054-023-04327-7 PMC9977110

[6] Shackelford SA, del Junco DJ, Powell-Dunford N, Mazuchowski EL, Howard JT, Kotwal RS, et al. Association of prehospital blood product transfusion during medical evacuation of combat casualties in Afghanistan with acute and 30-day survival. JAMA. 2017;318:1581. 10.1001/jama.2017.15097. 29067429 10.1001/jama.2017.15097 PMC5818807

[7] Shand S, Curtis K, Dinh M, Burns B. What is the impact of prehospital blood product administration for patients with catastrophic haemorrhage: an integrative review. Injury. 2019. 10.1016/j.injury.2018.11.049. 30578085 10.1016/j.injury.2018.11.049

[8] Thies KC, Truhlář A, Keene D, Hinkelbein J, Rützler K, Brazzi L, et al. Pre-hospital blood transfusion- a n ESA survey of European practice. Scand J Trauma Resusc Emerg Med. 2020. 10.1186/s13049-020-00774-1. 32795320 10.1186/s13049-020-00774-1 PMC7427720

[9] Selleng K, Baschin M, Henkel B, Jenichen G, Thies KC, Rudolph M, et al. Blood product supply for a helicopter emergency medical service. Transfus Med Hemother. 2021. 10.1159/000519825. 35082564 10.1159/000519825 PMC8740152

[10] Pusateri AE, Moore EE, Moore HB, Le TD, Guyette FX, Chapman MP, et al. Association of prehospital plasma transfusion with survival in trauma patients with hemorrhagic shock when transport times are longer than 20 minutes: a post hoc analysis of the PAMPer and COMBAT clinical trials. JAMA Surg. 2020. 10.1001/jamasurg.2019.5085. 31851290 10.1001/jamasurg.2019.5085 PMC6990948

[11] Tucker H, Brohi K, Tan J, Aylwin C, Bloomer R, Cardigan R, et al. Association of red blood cells and plasma transfusion versus red blood cell transfusion only with survival for treatment of major traumatic hemorrhage in prehospital setting in England: a multicenter study. Crit Care. 2023. 10.1186/s13054-022-04279-4. 36650557 10.1186/s13054-022-04279-4 PMC9847037

[12] Morgan KM, Abou-Khalil E, Strotmeyer S, Richardson WM, Gaines BA, Leeper CM. Association of prehospital transfusion with mortality in pediatric trauma. JAMA Pediatr. 2023;177:693. 10.1001/jamapediatrics.2023.1291. 37213096 10.1001/jamapediatrics.2023.1291 PMC10203962

[13] Gaessler H, Helm M, Kulla M, Hossfeld B, Riedel J, Kerschowski J, et al. Prehospital predictors of the need for transfusion in patients with major trauma. Eur J Trauma Emerg Surg. 2023. 10.1007/s00068-022-02132-5. 36222858 10.1007/s00068-022-02132-5 PMC10175474

[14] Gaessler H, Helm M, Kulla M, Hossfeld B, Riedel J, Kerschowski J, et al. Prehospital predictors of the need for transfusion in patients with major trauma. Eur J Trauma Emerg Surg. 2023;49:803–12. 10.1007/s00068-022-02132-5. 36222858 10.1007/s00068-022-02132-5 PMC10175474

[15] Sullivan TM, Milestone ZP, Tempel PE, Gao S, Burd RS. Development and validation of a Bayesian belief network predicting the probability of blood transfusion after pediatric injury. J Trauma Acute Care Surg. 2023;94:304–11. 10.1097/TA.0000000000003709. 35696359 10.1097/TA.0000000000003709 PMC9748028

[16] World Medical Association Declaration of Helsinki. JAMA. 2013;310:2191. 10.1001/jama.2013.281053. 24141714 10.1001/jama.2013.281053

[17] von Elm E, Altman DG, Egger M, Pocock SJ, Gøtzsche PC, Vandenbroucke JP. The strengthening the reporting of observational studies in epidemiology (STROBE) statement: guidelines for reporting observational studies. J Clin Epidemiol. 2008;61:344–9. 10.1016/j.jclinepi.2007.11.008. 18313558 10.1016/j.jclinepi.2007.11.008

[18] Chidester SJ, Williams N, Wang W, Groner JI. A pediatric massive transfusion protocol. J Trauma Acute Care Surg. 2012. 10.1097/TA.0b013e318265d267. 23064608 10.1097/TA.0b013e318265d267 PMC3821172

[19] Evangelista ME, Gaffley M, Neff LP. Massive transfusion protocols for pediatric patients: current perspectives. J Blood Med. 2020. 10.2147/JBM.S205132. 32547282 10.2147/JBM.S205132 PMC7247594

[20] Pape HC, Lefering R, Butcher N, Peitzman A, Leenen L, Marzi I, et al. The definition of polytrauma revisited: an international consensus process and proposal of the new ‘Berlin definition.’ J Trauma Acute Care Surg. 2014. 10.1097/TA.0000000000000453. 25494433 10.1097/TA.0000000000000453

[21] Lefering R, Huber-Wagner S, Nienaber U, Maegele M, Bouillon B. Update of the trauma risk adjustment model of the TraumaRegister DGU™: the revised injury severity classification, version II. Crit Care. 2014. 10.1186/s13054-014-0476-2. 25394596 10.1186/s13054-014-0476-2 PMC4177428

[22] Shroyer MC, Griffin RL, Mortellaro VE, Russell RT. Massive transfusion in pediatric trauma: analysis of the National Trauma Databank. J Surg Res. 2017;208:166–72. 10.1016/j.jss.2016.09.039. 27993204 10.1016/j.jss.2016.09.039

[23] Yin G, Radulovic N, O’Neill M, Lightfoot D, Nolan B. Predictors of transfusion in trauma and their utility in the prehospital environment: a scoping review. Prehosp Emerg Care. 2023;27:575–85. 10.1080/10903127.2022.2120935. 36066217 10.1080/10903127.2022.2120935

[24] Pommerening MJ, Goodman MD, Holcomb JB, Wade CE, Fox EE, del Junco DJ, et al. Clinical gestalt and the prediction of massive transfusion after trauma. Injury. 2015;46:807–13. 10.1016/j.injury.2014.12.026. 25682314 10.1016/j.injury.2014.12.026 PMC4800814

[25] Stevens J, Reppucci ML, Meier M, Phillips R, Shahi N, Shirek G, et al. Pre-hospital and emergency department shock index pediatric age-adjusted (SIPA) “cut points” to identify pediatric trauma patients at risk for massive transfusion and/or mortality. J Pediatr Surg. 2022. 10.1016/j.jpedsurg.2021.09.053. 34753559 10.1016/j.jpedsurg.2021.09.053

[26] Reppucci ML, Acker SN, Cooper E, Meier M, Stevens J, Phillips R, et al. Improved identification of severely injured pediatric trauma patients using reverse shock index multiplied by Glasgow Coma Scale. J Trauma Acute Care Surg. 2022. 10.1097/TA.0000000000003432. 34932042 10.1097/TA.0000000000003432

[27] Wohlgemut JM, Marsden MER, Stoner RS, Pisirir E, Kyrimi E, Grier G, et al. Diagnostic accuracy of clinical examination to identify life- and limb-threatening injuries in trauma patients. Scand J Trauma Resusc Emerg Med. 2023. 10.1186/s13049-023-01083-z. 37029436 10.1186/s13049-023-01083-z PMC10082501

[28] Hasler RM, Kehl C, Exadaktylos AK, Albrecht R, Dubler S, Greif R, et al. Accuracy of prehospital diagnosis and triage of a Swiss helicopter emergency medical service. J Trauma Acute Care Surg. 2012. 10.1097/TA.0b013e31825c14b7. 22929499 10.1097/TA.0b013e31825c14b7

[29] de Carvalho Panzeri Carlotti AP, do Amaral VH, de Carvalho Canela Balzi AP, Johnston C, Regalio FA, Cardoso MF, et al. Management of severe traumatic brain injury in pediatric patients: an evidence-based approach. Neurol Sci. 2025;46:969–91. 10.1007/s10072-024-07849-2. 39476094 10.1007/s10072-024-07849-2

