# Supplementary figures and images for: Indicators of early transfusion in paediatric trauma: a retrospective analysis of 11,849 cases from the TraumaRegister DGU®
Source: Scand J Trauma Resusc Emerg Med. 2025 Nov 26;33:195. doi: 10.1186/s13049-025-01516-x (PMC12676769; doi:10.1186/s13049-025-01516-x)

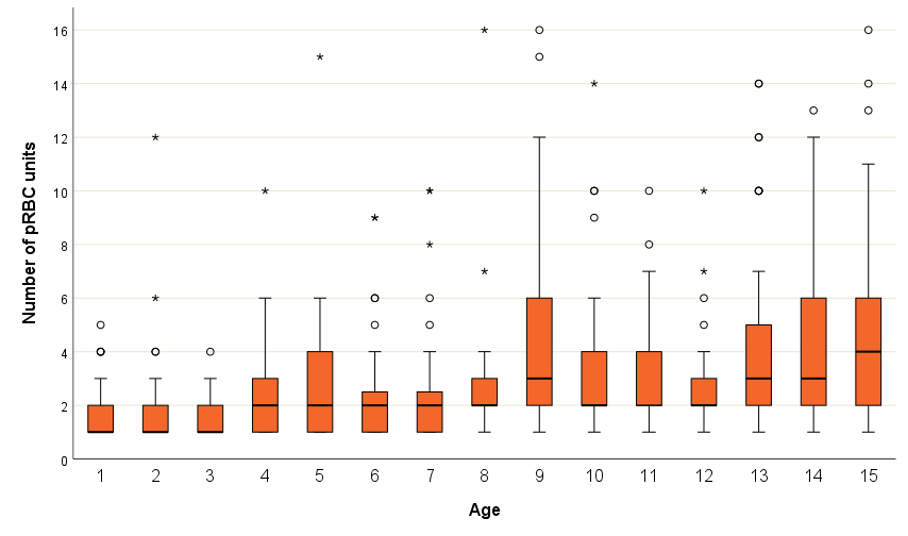

Supplement: Supplementary file 1 — Additional file 1. Supplemental Figure 1. Age distribution of cases with massive transfusion. Age is demonstrated as years. Abbreviations: pRBC: packed red blood cell concentrate [file 13049_2025_1516_MOESM1_ESM.png]
